# Validation of a RP-HPLC-DAD Method for Chamomile (*Matricaria recutita*) Preparations and Assessment of the Marker, Apigenin-7-glucoside, Safety and Anti-Inflammatory Effect

**DOI:** 10.1155/2015/828437

**Published:** 2015-09-03

**Authors:** Felipe Galeti Miguel, Amanda Henriques Cavalheiro, Nathália Favaretto Spinola, Diego Luis Ribeiro, Gustavo Rafael Mazzaron Barcelos, Lusânia Maria Greggi Antunes, Juliana Issa Hori, Franciane Marquele-Oliveira, Bruno Alves Rocha, Andresa Aparecida Berretta

**Affiliations:** ^1^Laboratório de Pesquisa, Desenvolvimento e Inovação (P, D & I), Apis Flora Industrial e Comercial LTDA, 14020-670 Ribeirão Preto, SP, Brazil; ^2^Departamento de Genética, Faculdade de Medicina de Ribeirão Preto, Universidade de São Paulo (FMRP-USP), 14055-370 Ribeirão Preto, SP, Brazil; ^3^Departamento de Análises Clínicas, Toxicológicas e Bromatológicas, Faculdade de Ciências Farmacêuticas de Ribeirão Preto, Universidade de São Paulo (FCFRP-USP), 14040-903 Ribeirão Preto, SP, Brazil; ^4^Department of Pharmacology, School of Medicine of Ribeirao Preto, University of Sao Paulo, 14055-370 Ribeirao Preto, SP, Brazil; ^5^Departamento de Ciências Farmacêuticas, Faculdade de Ciências Farmacêuticas de Ribeirão Preto, Universidade de São Paulo (FCFRP-USP), 14040-903 Ribeirão Preto, SP, Brazil

## Abstract

Chamomile is a medicinal plant, which presents several biological effects, especially the anti-inflammatory effect. One of the compounds related to this effect is apigenin, a flavonoid that is mostly found in its glycosylated form, apigenin-7-glucoside (APG), in natural sources. However, the affectivity and safety of this glycoside have not been well explored for topical application. In this context, the aim of this work was to develop and validate a reversed-phase high-performance liquid chromatography (RP-HPLC-DAD) method to quantify APG in chamomile preparations. Additionally, the safety and the anti-inflammatory potential of this flavonoid were verified. The RP-HPLC-DAD method was developed and validated with linearity at 24.0–36.0 *μ*g/mL range (*r* = 0.9994). Intra- and interday precision (RSD) were 0.27–2.66% and accuracy was 98.27–101.21%. The validated method was applied in the analysis of chamomile flower heads, glycolic extract, and Kamillen cream, supporting the method application in the quality control of chamomile preparations. Furthermore, the APG safety was assessed by MTT cytotoxicity assay and mutagenic protocols and the anti-inflammatory activity was confirmed by a diminished TNF-*α* production showed by mice macrophages treated with APG following LPS treatment.

## 1. Introduction

Chamomile (*Matricaria recutita* L.) is an annual, aromatic, and herbaceous plant from Asteraceae family, native to Southern and Eastern Europe and Western Asia [1, 2]. Several reports have demonstrated its cultivation in Europe, South America and, to a lesser extent, in Africa [3, 4]. Chamomile is one of the most commonly consumed herbal tea worldwide, and, in addition, not only it is an ingredient in several traditional and medicinal preparations, but it is also is employed in pharmaceutical and cosmetic industries [1, 5–7]. Its antimicrobial, antispasmodic, and anti-inflammatory properties have already been demonstrated [8–10] especially in dermatological application, in which the chamomile use presented cutaneous and mucosal inflammation reduction [4, 11, 12].

Regarding chemical composition, more than 120 chemical metabolites have been identified in chamomile, including phenolic compounds like flavonoids (apigenin, quercetin, patuletin, luteolin, and their glucosides), sesquiterpenes (*α*-bisabolol, bisabolol oxides A and B, chamazulene, and farnesene), coumarins, and several others [6, 7, 9].

Topical chamomile preparations are indicated for skin and mucosa inflammation and irritation and can be applied in any part of the body [9, 13, 14]. Around 3–10% w/w drug amount is recommended for topical formulation or equivalent preparations [9, 13, 14]. In this context, characterizing a reproducible and safe chamomile preparation marker presents great importance for industrial field.

The apigenin is a low-toxicity and nonmutagenic flavonoid [15] with anti-inflammatory actions [7, 16] and it is considered a suitable marker. Nevertheless, some considerations must be undertaken regarding its glucoside form depending on the administration route. In one hand, for oral route, chamomile preparations may present free or glucoside apigenin, once the digestive mammals tract can hydrolyze glucosides [17, 18]. On the other hand, chamomile flowers, usually employed in topical preparations as raw material, present glycosylated apigenin instead of free apigenin [5], which, therefore, requires the assessment of safety and anti-inflammatory properties of this marker for topical use.

Based on these aspects, a chamomile extract and a topical formulation for anti-inflammatory purposes (Kamillen) were developed and here we demonstrated their chemical analysis, as well as the marker biological activity. We showed not only the development and validation of a reliable, fast, and easy methodology for quantification of apigenin-7-glucoside (APG), but also the* in vitro* safety and efficacy of this flavonoid. Although other methodologies for chamomile assessment have been already previously published [5, 19], this is the first time that three distinct and complex matrixes were considered and added with the aim of future employment of this method in industry routine of quality control.

## 2. Materials and Methods

### 2.1. Chemicals and Reagents

Apigenin-7-glucoside was purchased from Sigma-Aldrich (St. Louis, MO, USA) and the purity was >98% as determined by HPLC. The HPLC-grade solvents, methanol and acetonitrile, were supplied by JT Backer (Mexico) and purified water was obtained using a Milli-Q Direct Q-5 filter system of Millipore (USA). Other reagents, such as acetic acid, sodium hydroxide, and hydrochloric acid were purchased from Synth (Brasil). Benzo(a)pyrene (B(a)P; CAS 50-32-8), and 3-(4,5-dimethylthiazol-2-yl)-2,5-diphenyltetrazolium bromide (MTT) were purchased from Sigma-Aldrich (St. Louis, MO, USA). Gel Red was obtained from Biotium (Hayward, CA, USA). Dulbecco's Modified Eagle Medium (DMEM) and fetal bovine serum (FBS) were purchased from Gibco (Carlsbad, CA, USA). Low melting point (LMP) agarose and normal melting point (NMP) agarose came from Invitrogen (California, CA, USA). All other chemicals were analytical grade products and were purchased from Sigma-Aldrich (St. Louis, MO, USA).

### 2.2. Materials and Samples Preparation

Five different commercial batches of floral heads of* M. recutita* were acquired by the authors, from Santos Flora Co. (São Paulo, Brazil). Chamomile glycolic extract and Kamillen cream were developed and supplied, two batches of each, by Apis Flora Ltda (Ribeirão Preto, Brazil). The 1.5 g of air-dried and powdered floral heads of the* M. recutita *was subjected to soxhlet extraction for 4 h with 100 mL ethanol 70%. Next, the ethanol extract was concentrated under reduced pressure. After ethanol extract concentration, sodium hydroxide 1.6% was added and submitted to ultrasonication for 30 min. Subsequently, the pH of the solution was corrected to 5.0 with a hydrochloric acid solution (50%). The volume of solution was completed to 100 mL with methanol. A 5 mL aliquot was diluted to 25 mL with methanol, homogenized and filtered through a 0.45 *μ*m membrane filter and, injected into HPLC (*drug extract*).

The chamomile glycolic extract was obtained with 1.5 g of air-dried and powdered floral heads of the* M. recutita* subjected to soxhlet extraction for 4 h with 100 mL ethanol 70%. Next, this extract was concentrated under reduced pressure and until complete solvent evaporation followed by propylene glycol addition. The drug : extract ratio was 1 : 1. For sample preparation analysis, 225 mg was weighed and added with 20 mL of sodium hydroxide (1.6%). This mixture was subjected to ultrasonication for 30 min and the pH of solution was corrected to 5.0 with a hydrochloric acid solution (50%). The volume of solution was completed to 100 mL with methanol. A 5 mL aliquot was diluted to 25 mL with methanol, homogenized and filtered through a 0.45 *μ*m membrane filter, and injected into HPLC (*glycolic extract*).

The Kamillen cream was obtained with the dispersion of chamomile glycolic extract, rose and lavender essential oil, almond and vitamin E oil, zinc oxide, vegetable glycerin, volatile silicon, and fomblin, in a hostacerin emulsion previously dispersed in microbiological conserved purified water (potassium sorbate 0.1%). For sample preparation, 4.4 g of Kamillen was weighed and added with 20 mL of sodium hydroxide (1.6%). This mixture was subjected to ultrasonication for 30 min. After this time, with a hydrochloric acid solution (50%), the pH of solution was corrected to 5.0. The volume of solution was completed to 50 mL with methanol, homogenized and filtered through a 0.45 *μ*m membrane filter, and injected into HPLC (*chamomile cream*).

### 2.3. Standard Solutions

Standard solutions were prepared in methanol : water (1 : 1) by dissolving the appropriate amount of APG (0.5 mg/mL), using an ultrasonic bath and vortex agitation, and stored below 4°C. Working standard solutions were prepared by serial dilution of stock solutions with methanol and water to achieve the final concentrations required for the calibration curve (24.0, 27.0, 30.0, 33.0, and 36.0 *μ*g/mL). Standard solutions were filtered through 0.45 *μ*m filter and injected in triplicate. The obtained curves were employed to validate the reproducibility of the method and for sample quantification.

### 2.4. Chromatographic Apparatus and Analytical Conditions

RP-HPLC-DAD method development and validation procedure was performed in an instrumentation consisting of a Shimadzu Liquid Chromatograph, LC-20AT quaternary delivery system, equipped with an SIL-20A autosampler, a CTO-10AC column oven, and a DAD-SPD-M20A photodiode array detector (Kyoto, Japan). Analytical conditions were optimized and a reverse-phase ShimPack CLC-ODS (C18) analytical column (250 mm × 4.6 i.d. and a particle size of 5 *μ*m) from Shimadzu (Tokyo, Japan), protected by a guard-column from the same stationary phase, was used. After the method development, the optimal conditions were as follows: mobile phase consisting of a gradient (Table 1) of purified water acidified with 0.05% of acetic acid, (Phase A) and acetonitrile (Phase B). The flow rate, detection wavelength, column oven temperature, and sample injection volume were 1.0 mL·min^−1^, 335 nm, 40°C, and 15 *μ*L, respectively. Peak was assigned by comparison with authenticated standard and based on the retention time and UV spectra under the same analytical conditions.

### 2.5. Method Validation

The method was validated according to the Brazilian rules for analytical method validation [20] and International Committee on Harmonization (ICH) guidelines [21]. In this case, the parameters evaluated included selectivity, linearity, precision (repeatability and intermediate precision), and matrix effect.

*Selectivity* was determined by analyzing the separation and resolution of the peak of samples and standard solutions of the APG. The ability of the method to distinguish the analyte among possible interferences was also assessed. Purity of peak and retention factor for APG working solution (30 *μ*g/mL) were evaluated by UV spectra (190–400 nm) recorded at different points of the chromatographic peak and in chromatograms of sample solutions.

*Linearity* was evaluated during three nonconsecutive days (*n* = 3) from points of calibration solutions (24.0, 27.0, 30.0, 33.0, and 36.0 *μ*g/mL) of APG. The linearity was determined by examining the correlation coefficient (*r*) of the linear regression line for the response versus concentration of the calibration curves prepared as described earlier.

*Precision* was estimated by evaluating the within-day (intraday, repeatability) and between-day (interday, intermediate precision) results of analyses carried out on two different and consecutive days. Both precision and accuracy tests were evaluated at one-concentration level and six replicates at 100% of the test concentration 30 *μ*g/mL. Precision of the assay was calculated as coefficient of variation, whereas the accuracy was calculated as the relative error of the mean, between expected and calculated concentrations. The accuracy was evaluated at three concentration levels within the linearity range: low (24.0 *μ*g/mL, 80%), medium (30.0 *μ*g/mL, 100%), and high (36.0 *μ*g/mL, 120%). The precision was evaluated from sample solutions analyzed in the same day (*n* = 6), whereas interday precision was accessed by analyzing sample solutions prepared in three different days by two different analysts (*n* = 6).

For* method robustness*, APG solution at 30 *μ*g/mL was analyzed employing the established conditions and under variations of flow rate (0.8 mL/min) and oven temperature (45°C). All parameters were evaluated in order to determine whether the method is robust after each variation, in terms of peak resolution, asymmetry, and concentration of the analytes.

The statistical analysis of the data obtained in the validation and stability studies was performed using Excel software, version 2007 (Microsoft).

### 2.6. Determination of TNF-*α* Levels in Mouse BMDMs

For cytokine determination, Bone Marrow Derived Macrophages (BMDMs) from C57BL/6 mice were prepared as previously described [22]. Briefly, bone marrow cells from femurs of adult mice were cultured for 7 days in RPMI 1640, containing 20% fetal bovine serum (FBS) and 30% L-929 cell conditioned media (LCCM).

Macrophages (2.0 × 10^5^) were plated in 24-well plates for 16 h at 37°C, 5% CO_2_ in RPMI 140 media containing 10% FBS and 5% of LCCM. Next, cells were treated with LPS from* Escherichia coli *(Sigma) at concentration of 1 *μ*g/mL during 4 hours for induction of the proinflammatory cytokine TNF-*α*. After this time, cells were treated or not with different concentrations of APG for additional 14 hours.

The supernatant was collected and the cytokine was measured by enzyme-linked immunosorbent assay (ELISA) with a mouse TNF-*α* kit (R&D Quantikine ELISA) according to the manufacturer's instructions.

### 2.7. Cell Culture Conditions and Treatment

HepG2 cells were kindly provided by Professor Dr. Luiz Gonzaga Toni (University of São Paulo, Brazil). Stock cultures of the cells (1.0 mL portions with 10^6^ viable cells in DMEM with 15% of FBS and 5.0% DMSO) were stored in liquid nitrogen. For the experiments described in this study, the cells were defrosted and used between the 3rd and 8th passages. In all experiments, the cells were cultivated for a complete cell cycle in DMEM with 15% FBS and 1.0% of penicillin and streptomycin for 24 hours in culture flasks (75 cm^2^; Corning, Lowell, MA, USA) in 5.0% CO_2_ atmosphere at 37°C and 95% relative humidity. For subcultivation, the cells were trypsinized, washed with phosphate buffered saline (PBS, pH 7.4), centrifuged (100 ×g, 5 min), and separated by pressing the suspensions through a syringe (needle 0.4 × 19, Becton Dickinson, São Paulo, Brazil).

To evaluate the acute toxic effects of the flavonoid on cell viability, the cells were treated with concentrations of APG as follows: 0.10, 0.50, 1.0, 5.0, 10, 50, and 100 *μ*g/mL during the period of 24 hours and, thereafter, the viability of the cells was assessed by MTT assay. To assess the impact of the treatments of APG on DNA stability by use of Comet Assay, the cells were exposed to different concentrations (0.10, 1.0, and 10 *μ*g/mL) during 4 hours. In all experiments, solvent controls were included (DMSO: 1.0%) as well as positive controls (benzo(a)pyrene (B(a)P): 10 *μ*g/mL).

### 2.8. Cell Viability Assay (MTT)

MTT assays were conducted as described by Mosmann [23]. Absorbances at 570 nm were measured on a microenzyme-linked immunosorbent assay (ELISA) reader (EL800, Gen5 Data Analysis Software, BioTek, Winooski, VT, USA). Independent cultures were made in parallel in three different 96-well microplates to evaluate cell viability. For each culture (microplate), the treatments were done in replicate. Results of the experiments are expressed as percentage of viable cells (% of viable cells).

### 2.9. Single Cell Gel Electrophoresis (SCGE) Assays

The SCGE experiments were carried out according to the protocol of Uhl et al. [24]. Briefly, 5.0 × 10^4^ cells were transferred to agarose-coated slides which were transferred after lysis to an electrophoresis chamber with buffer (300 mM NaOH and 1.0 mM EDTA, pH > 13). Electrophoresis was conducted under standard conditions (25 V; 300 mA; 1.25 V, cm^−1^) for 20 min, and subsequently the slides were neutralized, air-dried, and fixed in absolute ethanol.

The slides were stained with Gel Red and evaluated under a fluorescence microscope (Axiostar, Zeiss, Germany) with 40x magnification. In all experiments, three cultures were made in parallel; from each, one slide was made and 50 nucleoids were evaluated. Comets were scored using the software Comet Assay IV (Perceptive Instruments, Bury St Edmunds, UK); the percentage of DNA in tail was measured as a parameter of DNA damage. All experiments were carried out in agreement with the guidelines for SCGE assays published by Tice et al. [25].

### 2.10. Statistical Analyses

All data analyses were performed with the SPSS 20 Statistics software (IBM; Armonk, NY, USA). Results were reported as mean ± standard deviation (SD). The means of different experiments were compared using one-way ANOVA and Dunnett's test. *p* values ≤ 0.050 were considered as significant.

## 3. Results and Discussion

### 3.1. Validation Methodology and Samples Analysis

**Figure 1:**
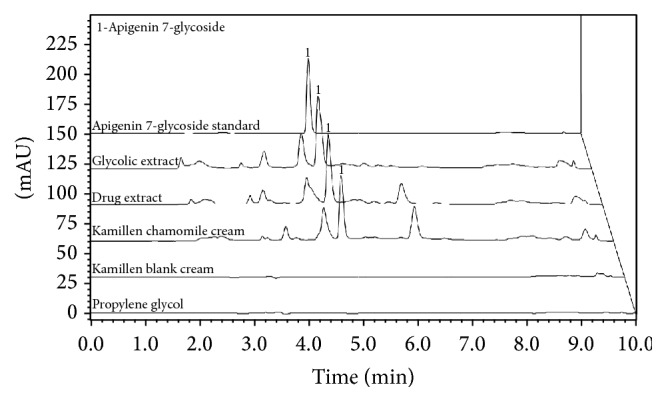
Comparison of RP-HPLC-DAD chromatograms of different chamomile samples and excipients used; propylene glycol; Kamillen blank cream; Kamillen chamomile cream; drug extract; glycolic extract; apigenin-7-glucoside standard.

The chromatographic separation optimization was mainly guided by assuring assay specificity and best separation time. Several mobile phase compositions and proportions were assessed and the set parameters were described in item “*Chromatographic Apparatus and Analytical Conditions.*” Acetic acid (0.05%) was added to improve peak shape and inhibit peak tailing. Flow rates were evaluated and 1 mL/min was set by promoting an optimum signal/noise ratio with reasonable separation time of 10 min. Based on the marker maximum UV absorption wavelength, 335 nm, and similar retention time, the APG peak was confirmed. Additionally, free apigenin was also assessed in order to demonstrate that the sample preparation procedure did not cause hydrolysis of the APG (data not shown). In summary, the proposed RP-HPLC-DAD methodology could suitably identify the standard in the samples (Figure 1).

The HPLC method validation was performed according to the ICH and Brazilian guidelines by assessing specificity, linearity, precision (repeatability and intermediate precision), recovery, and matrix effect [20, 21].

The selectivity of the method checked the presence of impurities or compounds in samples (drugs, glycolic extract, and Kamillen) that would interfere with the quantification and identification of APG based on retention time and wavelength absorption. Figure 1 showed the chromatograms with APG retention times from samples and standard solution (5 min) (Figure 1). The spectral profile analysis of APG peak in all samples evaluated demonstrated the methodology selectivity. No excipients employed in chamomile preparations, propylene glycol, and Kamillen blank cream interfered with the marker determination. The peak purity was checked for the standard and for all evaluated samples the results close to 1,000 factors were obtained at 335 nm. These results indicate that the peak presented little noise and no spectral coeluted impurities.

The method linearity was determined by assessing three analytical curves at five APG concentration levels (24.0–36.0 *μ*g/mL) (Table 2). The set concentrations comprised the required 80 to 120% range of the standard concentration used, attending Brazilian validation guidelines [20]. The APG calibration curve was linear over the established range (24.0–36.0 *μ*g/mL) with *r* of 0.9994 for the regression equation (*Y* = 387463*X* − 91511). Furthermore, the relative standard deviation for each concentration was lower than 0.41%, smaller than the maximum acceptable value (5.0%). Thus, we concluded that this method is linear in the selected range.

Intra- and interday variations (Table 3) were chosen to determine the precision of the developed method. The intraday variability test was performed using the same instrument and analyst at 30 *μ*g/mL of APG in sextuplicate. For drug plant, glycolic extract, and Kamillen cream, the assay was performed in the same way. The RSD values of the intraday test were found to be within the ranges of 0.38–2.66%, which was better when compared with intraday RSD% (2.69–3.05) obtained by Guzelmeric et al. [26], for this same compound using HPTLC methodology. However, Fonseca and Tavares [5] obtained 1.3% for this same parameter using capillary electrophoresis methodology. Interday precision was performed using the same instrumentation, but another analyst executed it in sextuplicate at 30 *μ*g/mL of APG. For plant drug, glycolic extract, and Kamillen cream, the assay was performed in the same way in sextuplicate. The RSD values of the interday test were found to be within the ranges of 0.27–2.44%. By comparing each average result for both analysts, we observed RSD values of 0.23, 1.47, 0.33, and 2.15% for APG standard, chamomile drug, extract, and cream, respectively. Our results were better than the previous one obtained with HPTLC technique [26] but lower than capillary electrophoresis (1.7%) [5].

The method accuracy (Table 4) was tested by three different concentrations (80%, 100%, and 120%) for APG solution and the samples presented accuracy values between 98.57 and 100.73%. Relative standard deviation values were lower than 5% for both intraday and interday assays, corroborating with accepted standards. The determined accuracy values were also within the criteria established by Anvisa guideline [20], which requires a range of 85 to 115% of nominal concentration. So, these results showed that all methodologies compared here presented very similar suitable recovery results [5, 26].

The RSD values found in robustness test showed no significant difference (<5.0%) in APG concentrations between experiments where variations in the analytical conditions established for the method were introduced. Both oven temperature and flow rate showed no influence in peak resolution, although asymmetry and time retention were affected. Hence, the results were shown to be robust thus indicating that small variations in the analytical conditions do not compromise the reliability of the results.

After properly validating the methodology developed, some commercial samples of chamomile head flowers acquired in the market and different batches of glycolic extracts and Kamillen cream were also evaluated. The results are presented in Table 5.

The results found in the chamomile head flowers, supplied in Brazilian market and collected during a year, showed some variations between the batches studies. APG amounts varied from 4.72 to 7.85 mg/g, in accordance with previous results of Mckay and Blumberg [4], and also with the content requested for United States Pharmacopoeia, which is at least 0.3% [19]. On the other hand, Guzelmeric et al. [26] evaluated chamomile head flowers from Istanbul and APG content was 1.90 ± 0.16 mg. Harbourne et al. [27] obtained 3.0 mg/g of APG for chamomile fresh flowers and around 2.0 mg/g for dried flowers (results expressed on dry basis). This marker decrease is acceptable since natural compounds are completely dependent on the dryness process of the flowers [27], solvent extraction used, cultivation geographic area, and soil conditions [5, 26].

The obtained results (Table 5) considering the APG content in glycolic extract (fluid extract 1 : 1) were in accordance with the chamomile flowers drug employed in the alcoholic extraction process, thus demonstrating a suitable extraction recovery. This high yield was obtained, since the exhaustive process was able to properly recover APG from the drug (6.66–7.01 mg/g for glycolic extracts). Data from literature shows that standardized extracts of chamomile contain 1.2% apigenin in 50% alcoholic extracts while aqueous extracts contain low concentrations of free apigenin but high levels of APG [2]. Harbourne et al. [27] obtained around 0.12% w/w yield using water as solvent (3.0 mg/g in an infusion from 2.5 g/100 mL).

Several compendiums recommend topical preparations to present 3–10% of the herbal drug (chamomile head flowers) for anti-inflammatory and skin damage treatment effects [9, 13, 14]. Queiroz et al. developed and evaluated “*in vivo*” safety and efficacy models regarding chamomile anti-inflammatory properties [28]. Gel preparations with 3% and 5% of chamomile dry matter, in the presence and absence of skin promoters, respectively, demonstrated anti-inflammatory effects similarly to sodium diclofenac cream [28]. Kamillen cream was developed considering this previous information (3–10% of drug in the formulation) and the results of APG content also corroborate with the fact that, during the industrial cream production, no marker loss happened and the recovery was in accordance with validation results.

### 3.2. Anti-Inflammatory Activity

*Matricaria recutita* head flowers were the plant selected in the present study due to its anti-inflammatory actions previously known [8–10]. Considering that APG is the major component of this plant [2, 5] and that a considerable number of studies have already demonstrated its anti-inflammatory effect as well, [7, 16], this study proposed evaluating the inhibition of TNF-*α* production by APG, the glycosylated presentation of apigenin, as an indicative parameter to consider the anti-inflammatory effect of this molecule.

**Figure 2:**
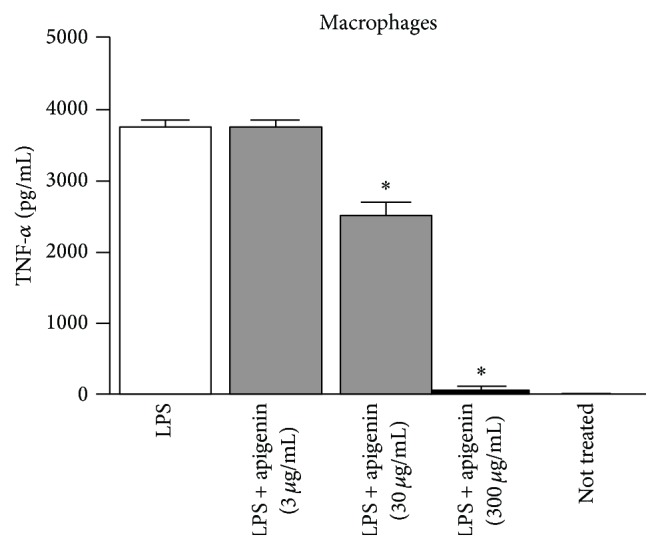
TNF-*α* production by mouse macrophages after treatment with apigenin-7-glucoside (APG). BMDMs cells were pretreated with LPS (1 *μ*g/mL) during 4 hours and then exposed to different concentrations of the flavonoid (3, 30, and 300 *μ*g/mL) for additional 12 hours. The TNF-*α* production was monitored in the cells supernatant by ELISA assay. Bars represent means ± SD of results obtained with triplicate samples. Asterisks indicate statistical significance between not treated and treated cells with the flavonoid (*p* ≤ 0.050; one-way ANOVA and Dunnett's test). “Not treated” indicates cells that were not treated with LPS.

The inhibition of TNF-*α* production by APG was assessed in LPS stimulated bone marrow macrophages from mice, a bona fide cell to study the responses of immune system. The results presented in Figure 2 showed a dose dependent inhibition of TNF-*α* production (30 and 300 *μ*g/mL) after the stimulation with LPS.

Bone marrow cell cultures produce IL-6 and TNF-*α* when stimulated by some stressors, especially lipopolysaccharide (LPS). The cytokine TNF-*α*, an important cytokine from innate immune response, was chosen due to its key role in the inflammatory response [22, 29]. Smolinski and Pestka [29] studied the production of IL-6 and TNF-*α*, in both* in vitro* and* in vivo* models, using LPS as a proinflammatory cytokine inducer and they demonstrated that apigenin, in the concentration of 0.1–10 *μ*g/mL, during 12 hours in murine macrophage cell culture exposition, inhibited the production of IL-6 but not TNF-*α*. On the other hand, pretreatment of animals with 50 mg/kg of APG, p.o., demonstrated the inhibition of both cytokines when the animals were prestimulated with LPS [29]. Probably, the dosage employed in the first* in vitro* assay was too low to demonstrate effectiveness. In the present study, we used 30–300 *μ*g/mL and we observed a considerable inhibition of TNF-*α* production. Moreover, the higher water solubility of APG when compared with free apigenin could have favored the results obtained here [2]. Of note, the results presented here are also in accordance with Krol et al. [30]. This group assessed inhibitors of photon emission on luminol-dependent chemiluminescence of neutrophils in in vitro model and they observed that among the ones with high activity, the glycosides were present, especially APG and [22] apiin.

These results suggest that APG obtained by our methodology can be considered a reliable marker for quality control of* M. recutita* since it still continues presenting characteristics already supported by the literature such as their anti-inflammatory activity.

### 3.3. Cell Viability Assay

**Figure 3:**
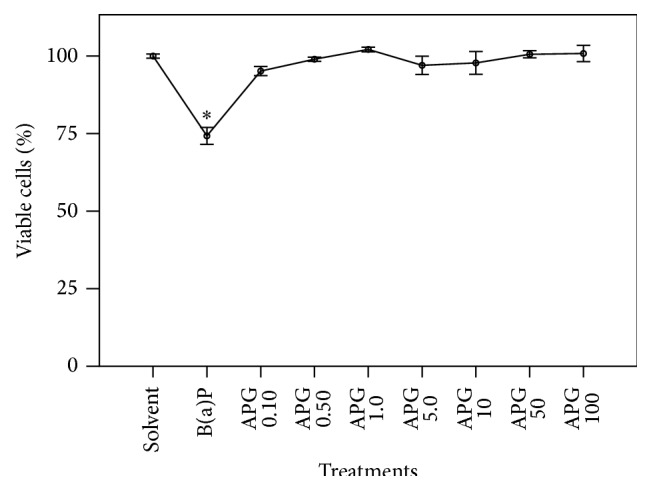
Viability of HepG2 cells after treatment with apigenin-7-glucoside (APG). The cultures were exposed to different concentrations of the flavonoid (0.10, 0.50, 1.0, 5.0, 10, 50, and 100 *μ*g/mL) during 24 hours. The percentage of cell viability was monitored by MTT assay. Bars represent means ± SD of results obtained with triplicate samples. Asterisks indicate statistical significance between B(a)P-treated cultures and cultures treated with the flavonoid (*p* ≤ 0.050; one-way ANOVA and Dunnett's test).

With respect to the safety studies, Figure 3 depicts the effects of several APG treatments on the cell viability using HepG2 cells as a model. It can be observed that the APG flavonoid did not cause any cytotoxic effects in all doses tested. In a previous study, Choi et al. [31] showed that concentrations of the flavonoid higher than 1.0 *μ*M (approximately 400 ng/mL) were able to decrease the cell viability in HepG2 cells. The differences observed between the study of Choi et al. [31] and ours may be explained by the apigenin glycosylation. At the present work, APG was assessed, while in the previous work the authors evaluated the free apigenin. This difference can be explained by the fact that flavonoids bounded to sugars present lower toxicity, in either* in vitro *and* in vivo *models [32]

**Figure 4:**
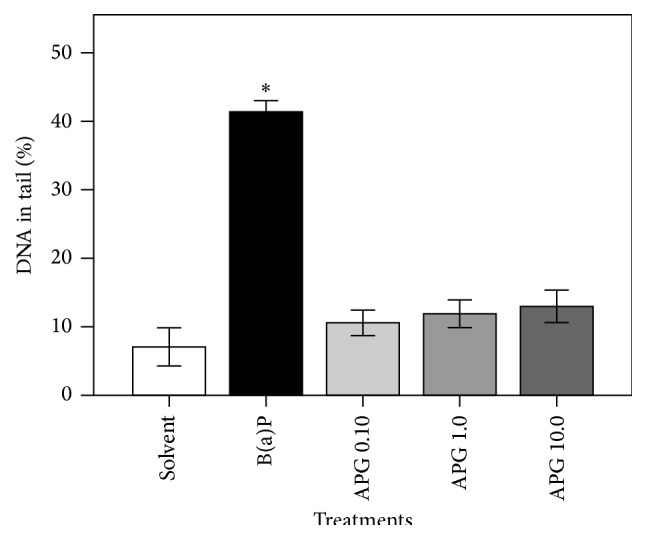
Evaluation of DNA stability in HepG2 cells after treatment with apigenin-7-glucoside (APG). The cultures were exposed to different concentrations of the flavonoid (0.10, 1.0, and 10 *μ*g/mL) during 4 hours. Subsequently, the DNA migration was assessed by the Comet Assay. Bars represent means ± SD of results obtained with triplicate samples. Asterisks indicate statistical significance between B(a)P-treated cultures and cultures treated with the flavonoid (*p* ≤ 0.050; one-way ANOVA and Dunnett's test).

Besides cytotoxicity assay by MTT method we also evaluate the impact of the treatments of APG on DNA stability, assessed by the SCGE assay. We observed that only the highest concentration of APG, as 10 *μ*g/mL, was able to increase the DNA migration of the cells (Figure 4). This observation may be explained, at least partly, regarding the flavonoid amount. It has been previously reported that flavonoids at high concentrations may act as prooxidants. Therefore, they can cause disturbances in the redox status of the cells and, consequently, increase the formation of reactive species, thus inducing DNA damage, as seen in the present study (for a comprehensive review, see Cemeli et al. [33]).

## 4. Conclusion

In conclusion, this study established a novel, simple, fast, and accurate reversed-phase high-performance liquid chromatography-photodiode array detection method for apigenin-7-glucoside determination in chamomile preparations. The method was validated according to Brazilian guidelines and showed suitable selectivity, linearity, precision, repeatability, robustness, and recovery with analysis time within 10 min. The proposed method could be employed for the quality control of chamomile plant extracts and other related preparations from this species. Besides this, another main point of this study was demonstrating not only the apigenin-7-glycoside applicability in chamomile characterization, but also its importance regarding chamomile biological activity. Therefore, the present work also demonstrates that apigenin-7-glycoside is safe and presents anti-inflammatory properties as demonstrated by the inhibition of TNF-*α* cytokine production in macrophages that were previously treated with LPS. To conclude, the safety and efficacy results found for this marker may also suggest that Kamillen cream has potential to be used as an anti-inflammatory product in skin disorders as rash of children.

## Figures and Tables

**Table 1:** Gradient elution used in the methodology.

Time (minutes)	Solvent (%)	
	Phase A	Phase B
0.01	74	26
1.00	58	42
4.50	56	44
4.80	10	90
6.00	10	90
6.50	74	26
8.00	74	26
10.01	74	26

**Table 2:** Linearity presentation for apigenin-7-glucoside in the range of 24.0 to 36.0 *μ*g/mL (*r* ^2^ = 0.9994, *n* = 3).

Concentration (*µ*g/mL)	Area			Average	%CV
	1	2	3		
24.00	834732	837400	836949	836360	0.17
27.00	968508	966327	960893	965243	0.41
30.00	1063463	1057985	1064616	1062021	0.33
33.00	1182345	1188481	1186655	1185827	0.27
36.00	1302084	1302401	1305261	1303249	0.13

**Table 3:** Intra- and interday repeatability presentation for apigenin 7-glucoside standard and chamomile drug, extract, and cream (*n* = 6).

Samples	Parameters			
	Area	%RSD	Concentration (*µ*g/mL)	%RSD
Repeatability				
Standard	1059493	0.41	29.65	0.38
Drug	1057617	2.86	29.60	2.66
Extract	1137418	0.94	31.68	0.88
Cream	1113728	0.62	31.07	0.58

Interday				
Standard	1062089	0.29	29.75	0.27
Drug	1079774	2.60	30.22	2.44
Extract	1140878	1.10	31.83	1.04
Cream	1148719	1.37	32.03	1.30

**Table 4:** Accuracy presentation results for chamomile drug, extract, and cream for low, medium, and high level concentrations (*n* = 3).

Level	Sample	Area	%RSD	Conc.	%RSD	Recovery	%RSD
Low	Drug	826626	0.33	23.66	0.31	98.57	0.31
	Extract	833495	0.64	23.90	0.59	99.57	0.59
	Cream	832876	0.35	23.86	0.32	99.53	0.32

Medium	Drug	1060745	0.76	29.86	0.72	99.52	0.72
	Extract	1050590	0.10	29.56	0.09	98.53	0.09
	Cream	1075772	0.78	30.22	0.72	100.73	0.72

High	Drug	1272652	0.52	35.47	0.50	98.53	0.50
	Extract	1279214	0.24	35.53	0.22	98.68	0.22
	Cream	1288514	0.39	35.79	0.37	99.41	0.37

**Table 5:** Apigenin-7-glucoside content in different batches of chamomile flower heads, glycolic extract, and Kamillen cream (*n* = 3) mg/g.

Samples	Apigenin-7-glucoside content (mg/g)
Drug	
CAFL 01/0413	7.30 ± 0.0869
CAFL 01/0513	7.85 ± 0.1098
CAFL 01/0613	4.72 ± 0.0358
CAFL 01/1013	6.28 ± 0.0473
17063072013	5.10 ± 0.0696

Glycolic extract	mg/g
Batch 17070113	6.66 ± 0.0786
Batch Exp	7.01 ± 0.0711

Kamillen cream	mg/g
Batch 17080112	0.396 ± 0.0025
Batch 17080212	0.358 ± 0.0025

## References

[1] Sebai H., Jabri M.-A., Souli A. (2014). Antidiarrheal and antioxidant activities of chamomile (*Matricaria recutita* L.) decoction extract in rats. *Journal of Ethnopharmacology*.

[2] Srivastava J. K., Shankar E., Gupta S. (2010). Chamomile: a herbal medicine of the past with a bright future. *Molecular Medicine Reports*.

[3] Arruda J., Approbato F., Maia M., Silva T., Approbato M. (2013). Efeito do extrato aquoso de camomila (*Chamomilla recutita* L.) na prenhez de ratas e no desenvolvimento dos filhotes. *Revista Brasileira de Plantas Medicinais*.

[4] McKay D. L., Blumberg J. B. (2006). A review of the bioactivity and potential health benefits of chamomile tea (*Matricaria recutita* L.). *Phytotherapy Research*.

[5] Fonseca F. N., Tavares M. F. M. (2004). Validation of a capillary electrophoresis method for the quantitative determination of free and total apigenin in extracts of *Chamomilla recutita*. *Phytochemical Analysis*.

[6] Guimarães R., Barros L., Dueñas M. (2013). Infusion and decoction of wild German chamomile: bioactivity and characterization of organic acids and phenolic compounds. *Food Chemistry*.

[7] Petronilho S., Maraschin M., Coimbra M. A., Rocha S. M. (2012). *In vitro* and *in vivo* studies of natural products: a challenge for their valuation. The case study of chamomile (*Matricaria recutita* L.). *Industrial Crops and Products*.

[8] British Herbal Medicine Association (1996). *British Herbal Pharmacopeia*.

[9] Man M.-Q., Hupe M., Sun R., Man G., Mauro T. M., Elias P. M. (2012). Topical apigenin alleviates cutaneous inflammation in murine models. *Evidence-Based Complementary and Alternative Medicine*.

[10] Loggia R. D., Carle R., Sosaand S., Tubaro A. (1990). Evaluation of the anti-inflammatory activity of Chamomile preparations. *Planta Medica*.

[11] Tubaro A., Zilli C., Redaelli C., Della Loggia R. (1984). Evaluation of antiinflammatory activity of a chamomile extract after topical application. *Planta Medica*.

[12] Jarrahi M. (2008). An experimental study of the effects of *Matricaria chamomilla* extract on cutaneous burn wound healing in albino rats.. *Natural Product Research*.

[13] Blumenthal M., Goldberg A., Brinckmann J. (2000). Herbal medicine. *Integrative Medicine Communications*.

[14] Bradley P. R. (1992). *British Herbal Compendium*.

[15] Nader N., Esmaeili S., Naghibi F., Mosaddegh M. (2006). HPTLC determination of apigenin in *Matricaria chamomilla* products. *Journal of Planar Chromatography*.

[16] Tomić M., Popović V., Petrović S. (2014). Antihyperalgesic and antiedematous activities of bisabolol-oxides-rich matricaria oil in a rat model of inflammation. *Phytotherapy Research*.

[17] Griffiths L. A., Smith G. E. (1972). Metabolism of apigenin and related compounds in the rat. Metabolite formation *in vivo* and by the intestinal microflora *in vitro*. *Biochemical Journal*.

[18] Schreiber A., Carle R., Reinhard E. (1990). On the accumulation of apigenin in chamomile flowers. *Planta Medica*.

[19] United States Pharmacopeia Dietary supplements—Botanicals.

[20] Agência Nacional de Vigilância Sanitária (ANVISA) (2003). *Guia para Validação de Métodos Analíticos e Bioanalíticos*.

[21] International Conference on Harmonization (ICH)

[22] Marim F. M., Silveira T. N., Lima D. S., Zamboni D. S. (2010). A method for generation of bone marrow-derived macrophages from cryopreserved mouse bone marrow cells. *PloS ONE*.

[23] Mosmann T. (1983). Rapid colorimetric assay for cellular growth and survival: application to proliferation and cytotoxicity assays. *Journal of Immunological Methods*.

[24] Uhl M., Helma C., Knasmüller S. (1999). Single-cell gel electrophoresis assays with human-derived hepatoma (Hep G2) cells. *Mutation Research*.

[25] Tice R. R., Agurell E., Anderson D. (2000). Single cell gel/comet assay: guidelines for *in vitro* and *in vivo* genetic toxicology testing. *Environmental and Molecular Mutagenesis*.

[26] Guzelmeric E., Vovk I., Yesilada E. (2015). Development and validation of an HPTLC method for apigenin 7-*O*-glucoside in chamomile flowers and its application for fingerprint discrimination of chamomile-like materials. *Journal of Pharmaceutical and Biomedical Analysis*.

[27] Harbourne N., Jacquier J. C., O'Riordan D. (2009). Optimisation of the extraction and processing conditions of chamomile (*Matricaria chamomilla* L.) for incorporation into a beverage. *Food Chemistry*.

[28] Queiroz M. B. R., Marcelino N. B., Ribeiro M. V., Espindola L. S., Cunha F. R., Da Silva M. V. (2009). Development of gel with Matricaria recutita L. extract for topic application and evaluation of physical-chemical stability and toxicity. *Latin American Journal of Pharmacy*.

[29] Smolinski A. T., Pestka J. J. (2003). Modulation of lipopolysaccharide-induced proinflammatory cytokine production *in vitro* and *in vivo* by the herbal constituents apigenin (chamomile), ginsenoside Rb_1_ (ginseng) and parthenolide (feverfew). *Food and Chemical Toxicology*.

[30] Krol W., Shani J., Czuba Z., Scheller S. (1992). Modulating luminol-dependent chemiluminescence of neutrophils by flavones. *Zeitschrift für Naturforschung C*.

[31] Choi S. I., Jeong C. S., Cho S. Y., Lee Y. S. (2007). Mechanism of apoptosis induced by apigenin in HepG2 human hepatoma cells: involvement of reactive oxygen species generated by NADPH oxidase. *Archives of Pharmacal Research*.

[32] Chao S.-C., Huang S.-C., Hu D.-N., Lin H.-Y. (2013). Subtoxic levels of apigenin inhibit expression and secretion of VEGF by uveal melanoma cells via suppression of ERK1/2 and PI3K/AKT pathways. *Evidence-Based Complementary and Alternative Medicine*.

[33] Cemeli E., Baumgartner A., Anderson D. (2009). Antioxidants and the Comet assay. *Mutation Research—Reviews in Mutation Research*.

